# Predictive modelling of knee osteoporosis

**DOI:** 10.1186/s13104-025-07125-2

**Published:** 2025-03-16

**Authors:** M. Siddharth, Gautam Arora, M. P. Vani

**Affiliations:** Information Technology, Vellore Institute of Technology, Vellore, Tamil Nadu India

**Keywords:** Osteoporosis, Random Forest, Predictive model, Machine learning

## Abstract

**Objective:**

The objective of this research was to develop a machine learning-based predictive model for osteoporosis screening using demographic and clinical data, including T-scores derived from calcaneus Quantitative Ultrasound (QUS). The study aimed to offer a cost-effective and accessible alternative to Dual-Energy X-ray Absorptiometry (DXA) scans, especially in resource-constrained settings.

**Results:**

The model achieved a classification accuracy of 88%, outperforming traditional decision trees by 10%. This improvement in accuracy demonstrates the potential of the random forest algorithm in identifying patients at risk of osteoporosis. Misclassification rates were minimal, with most errors occurring in distinguishing osteopenia from normal cases. The findings indicate that machine learning models trained on QUS data can aid in early identification of osteoporosis, reducing reliance on costly DXA scans.

## Introduction

Osteoporosis is a condition marked by diminished bone strength, disproportionately affecting older populations worldwide [1, 2]. Approximately 5% of men and 19% of women over 50 years old suffer from osteoporosis, totalling over 480 million individuals globally. The disease contributes to more than 9 million fractures annually, equating to one fracture every 2.8 s. In 2010, the economic burden of osteoporosis in the United States was estimated at $19 billion, with projections indicating this figure will exceed $28 billion by 2030. In Europe, the costs reached approximately $37 billion, while in countries like India and South Africa, the growing number of aging populations is expected to significantly increase osteoporosis-related healthcare expenses in the coming decades [3, 4].

Traditionally, osteoporosis is diagnosed through the measurement of Bone Mineral Density (BMD) using DXA, which has long been the gold standard for assessing bone health [5, 6]. However, DXA scans are costly and require specialized equipment and trained personnel, making them less accessible in low- and middle-income countries. To address this limitation, calcaneus QUS has emerged as an alternative method to estimate T-scores, offering a more affordable, portable, and accessible option compared to DXA. The World Health Organization (WHO) classification based on T-scores remains the same, with − 1.0 or higher considered normal, -1.0 to -2.5 indicating osteopenia, and below − 2.5 diagnostic of osteoporosis [7].

The T-Score from QUS can thus be incorporated to build large-scale screening programs aimed at identifying individuals at risk of osteoporosis, particularly in regions where DXA access is limited [8, 9]. Given these constraints, there is an increasing emphasis on identifying risk factors for osteoporosis early and implementing preventive strategies [10]. Early detection enables timely intervention, potentially reducing the occurrence of osteoporotic fractures and lowering the economic burden on healthcare systems.

Machine Learning (ML) has emerged as a promising tool in screening for osteoporosis by analysing patient health data without the need for expensive medical imaging. Recent studies have demonstrated the effectiveness of ML algorithms in identifying key risk factors, such as age, Body Mass Index (BMI), and lifestyle factors [2], which can predict low BMD with high accuracy. These advancements in ML-based models, coupled with QUS-derived T-scores, offer a potential solution to overcome the barriers associated with traditional diagnostic methods like DXA, making osteoporosis screening more accessible and cost-effective across diverse healthcare settings [8, 9].

Incorporating the QUS-estimated T-score in ML models further enhances the capabilities of such approaches. First, the use of the T-score from QUS enables enhanced screening and pre-diagnosis, particularly in regions where DXA is not accessible, allowing for more targeted recommendations for DXA testing in high-risk individuals. Secondly, osteoporosis is a multifactorial disease and combining QUS-based T-scores with other features such as age, BMI, and lifestyle factors improves the model’s predictive power, providing a more comprehensive risk assessment. Furthermore, historical T-scores, whether derived from QUS or DXA, can be used in the model to track progression over time, offering better insights into bone health trends. The inclusion of the T-score also serves as a validation measure, aligning the model’s predictions with established clinical criteria and ensuring robustness. Finally, the QUS-estimated T-score can reveal nuanced relationships in combination with other risk factors, contributing to more personalized and precise osteoporosis risk stratification.

## Methods and material

### Study design and data source

This study was designed to develop and validate a machine learning model for predicting the risk of osteoporosis. The data used for the model originates from the **Knee X-ray Osteoporosis Database**, an open-source dataset hosted on **Mendeley Data** [11]. This dataset includes 3210 patient records, focusing on individuals with knee-related issues, such as arthritis and osteoporosis. Each entry contains **demographic details** (e.g., age, gender), **clinical history** (e.g., menopause age, diabetes, thyroidism, fracture history), and **lifestyle factors** that contribute to bone health [11].

Unlike traditional methods relying on DXA scans, the dataset uses **QUS** to estimate the **T-score**, offering a more affordable and accessible diagnostic approach. The T-score is calculated based on ultrasound measurements from the knee joint, making this data particularly useful for models targeting populations with limited access to advanced imaging techniques.

This dataset provides a comprehensive array of features for machine learning, allowing the model to incorporate not only clinical and demographic data but also **longitudinal health records** and **fracture histories**, enhancing the prediction of osteoporosis risk across various healthcare settings.

### Data preparation

Patient data was randomly split into training data and test data (65% and 35% respectively) using a random sampling method available in in the scikit-learn library. This approach helps create an unbiased distribution of data for both training and testing, allowing the model to be evaluated on unseen data to assess its generalization performance.

Label encoding was performed to assign unique numerical values to categorical variables including but not limited to ‘Occupation’, ‘History of Fracture’, ‘Dialysis’ and ‘Family History of Osteoporosis’.

Alternatively, frequency encoding was used to encode variables such as ‘Daily Eating habits’, ‘Medical History’ and ‘Obesity’.

To address the imbalance of classes in the target variable, namely the ‘Diagnosis’ variable, which contained 149 patients diagnosed with Osteopenia, as opposed to 35 and 47 patients diagnosed with Normal Bone Density and Osteoporosis respectively, synthetic minority over sampling was applied on the training set, after the data splitting. This avoids data leakage, as the test set remains entirely unseen during the training process. By generating synthetic samples solely within the training set, we ensured the model’s evaluation on an independent test set, providing a more realistic measure of the model’s generalization ability. This generated about 312 data samples overall. This method involves generating new samples by finding nearest neighbours of minority class instances and creating new instances, that are combinations of these neighbours, improving the model’s ability to generalize data.

The random sampling method was chosen to maintain an unbiased division of the dataset. While small and imbalanced datasets could risk under-representing minority classes in either split, we addressed this by monitoring the class distribution post-split.

### Feature selection

**Fig. 1:**
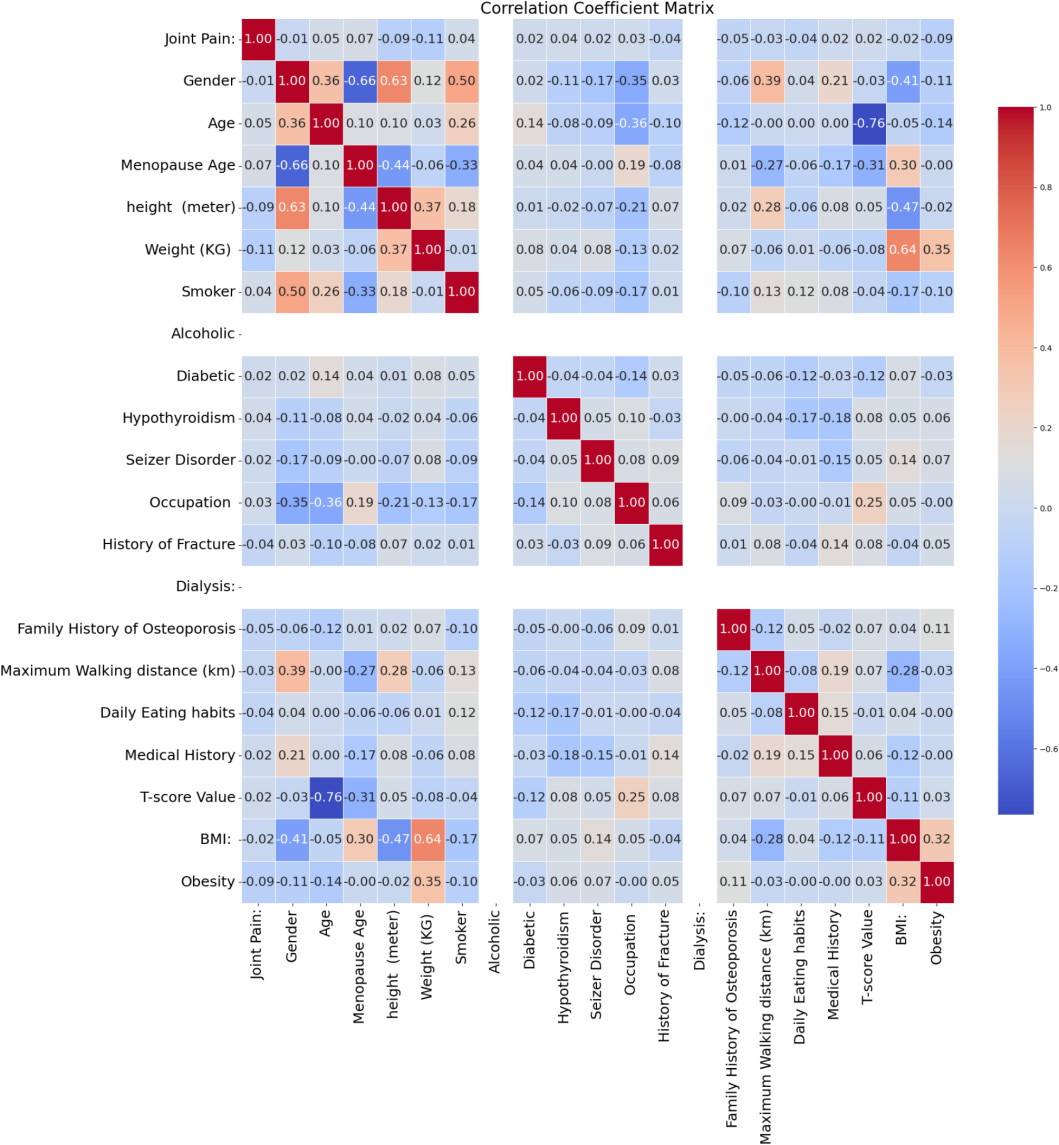
Correlation coefficient matrix

In the data preprocessing phase of this study, Pearson’s correlation coefficient was used to assess the relationships between various features. Based on the heatmap generated from the analysis, as depicted in Fig. 1, several pairs of columns exhibited high degrees of correlation, suggesting that one column from each pair could be dropped to reduce multicollinearity and enhance model efficiency. Specifically, the pairs of features were identified to be ‘Menopause Age’ and ‘Number of Pregnancies’, ‘T-score Value’ and ‘Z-Score Value’ and ‘Oestrogen Use’ and ‘Seizer Disorder’. Furthermore, certain unnecessary columns such as Site of Examination were dropped.

### Model deployment

Four machine learning models—XGBoost, AdaBoost, Decision Tree, and Random Forest [12] —were deployed and tested to evaluate their effectiveness in classifying patients using the provided clinical data. These models were initially evaluated without any prior hyperparameter tuning to assess their baseline performance. The results indicated that both XGBoost and AdaBoost exhibited signs of overfitting. Specifically, these models showed a large gap between their performance on the training data and their generalization to the test set.

On the other hand, the Decision Tree algorithm, while less prone to overfitting, achieved a relatively low accuracy score of 78%. In contrast, the Random Forest classifier showed promise, achieving a baseline accuracy score of 88%, indicating better generalization and performance compared to the other models.

## Results and discussion

All paragraphs must be indented. All paragraphs must be justified, i.e. both left-justified and right-justified.

### Model validation

**Fig. 2:**
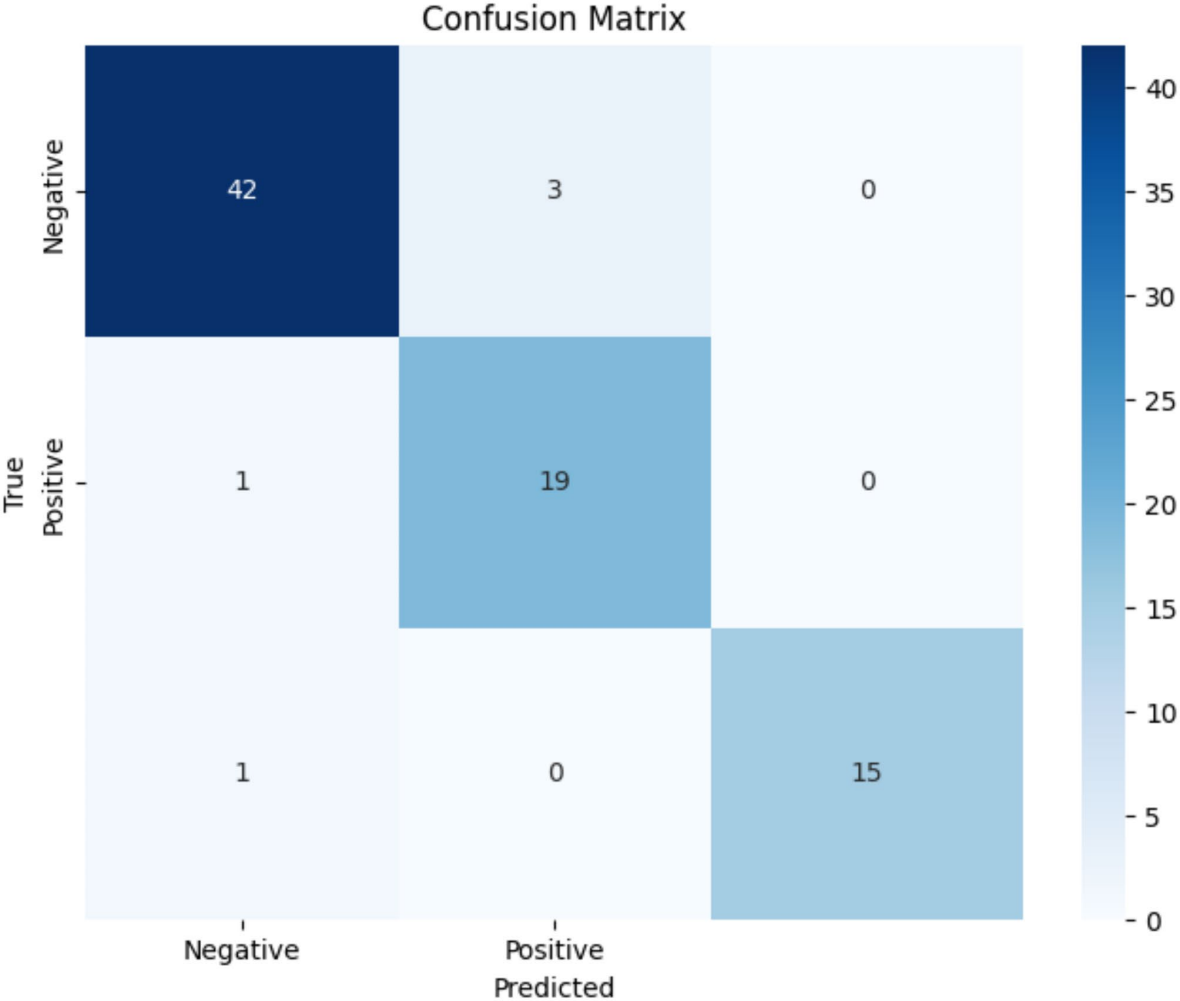
Confusion matrix

The model’s performance was first assessed using a confusion matrix, as depicted in Fig. 2. This revealed a classification accuracy of **93.8% post hyperparameter tuning**. The matrix indicated 19 true positives, **3** false positives, **1** true negative, and **42** false negatives, illustrating the model’s strengths and areas for improvement in predicting the various classes.

#### Normal classifications

The model correctly classified 42 individuals as having normal bone health, with 3 misclassified as osteopenia and none as osteoporosis.

#### Osteopenia classifications

The model accurately identified 19 individuals as having osteopenia, with 1 misclassified as normal and none as osteoporosis.

#### Osteoporosis classifications

The model successfully predicted 15 individuals as having osteoporosis, with 1 misclassified as normal and none as osteopenia.

### Feature importance

In our study, there is no single feature that drastically impacts the accuracy of the model. Instead, the Random Forest algorithm relies on a combination of features to achieve high classification performance in terms of diagnosis. A doctor, for instance, wouldn’t base their decision on just one exam; they would consider a variety of clinical assessments before concluding whether a patient is healthy or suffering from a condition like osteoporosis.

Similarly, in our model, while the T-score emerged as the most influential feature, it alone was not enough to classify patients accurately. The integration of other demographic and clinical factors, such as age, BMI, and menopause status, was crucial in the model’s decision-making process. These factors reflect broader health indicators that collectively provide a more complete picture of the patient’s bone health. This mirrors the multifaceted approach that healthcare professionals take, combining various sources of patient data to assess overall risk.

Moreover, by incorporating the T-score, a clinically established marker for bone density, our model captures a critical aspect of bone health, akin to a direct clinical index of bone strength. However, it is important to note that this marker is not a standalone determinant. The model’s ability to classify patients is reinforced by combining this feature with others, leading to more robust predictions. This collective use of features enhances the predictive accuracy in a way that simulates how different clinical test results collectively inform a diagnosis.

While the Random Forest classifier demonstrated a notable improvement in classification accuracy (88%) over the Decision Tree model (78%), the increase in complexity and computational demands raises concerns about interpretability—an essential factor in clinical settings. Although Random Forest models are harder to dissect compared to individual Decision Trees, they offer a comprehensive ranking of feature importance, shedding light on the most influential predictors.

**Fig. 3:**
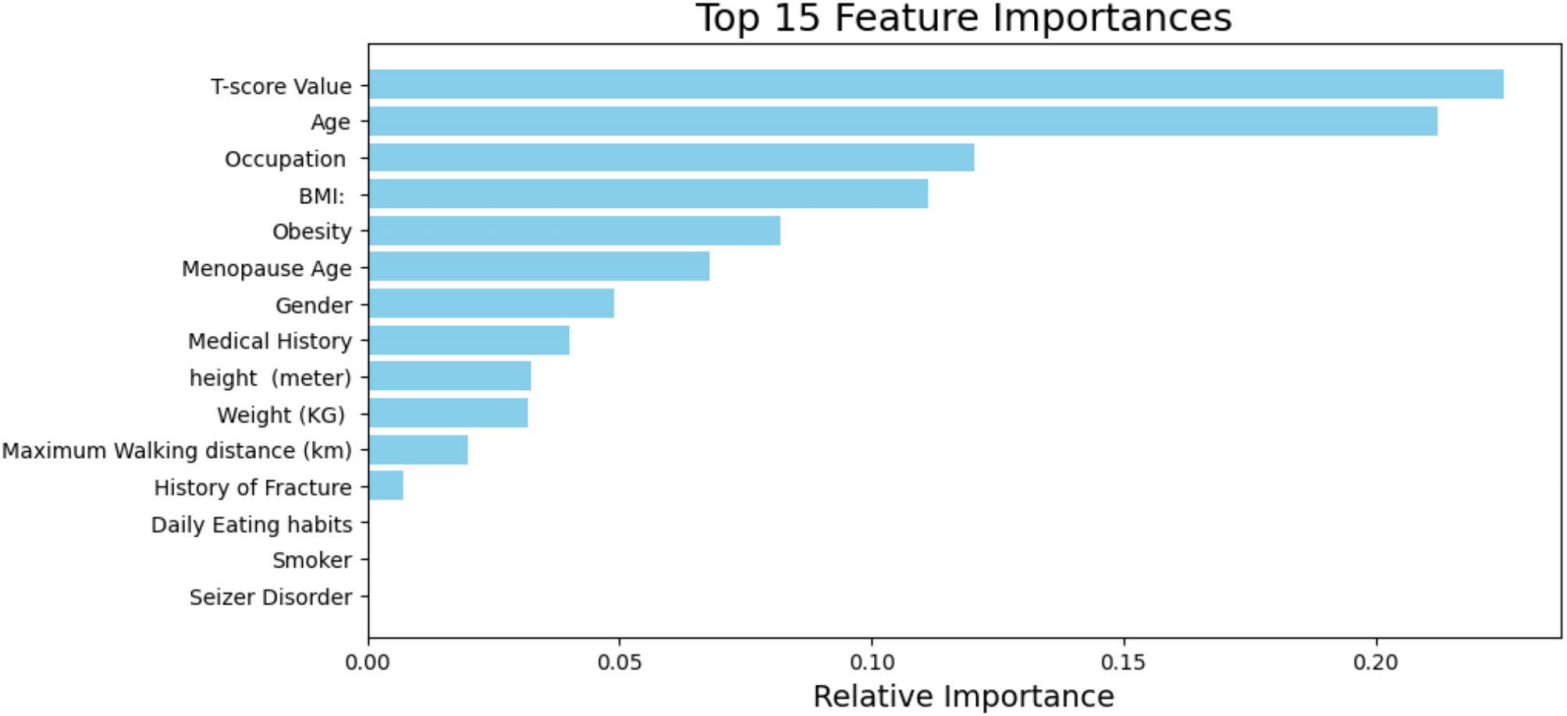
Relative importance of the considered features

In this study, the T-score emerged as the most critical variable, alongside BMI, age, and menopause status (Fig. 3). This ensemble approach provides robustness against overfitting and enhances predictive performance, which is advantageous for handling imbalanced datasets. However, for clinical deployment, simplicity and transparency are paramount. If statistical analysis reveals that the observed 10% performance gain is not significant, opting for a pruned Decision Tree could ensure model transparency while maintaining adequate accuracy.

Random Forest’s ability to process large volumes of complex data while highlighting critical variables provides valuable insights. Nevertheless, ongoing assessment of the statistical significance of performance improvements is essential to balance model accuracy and interpretability, ensuring the model’s applicability to real-world clinical scenarios.

### Demographic characteristics

**Fig. 4:**
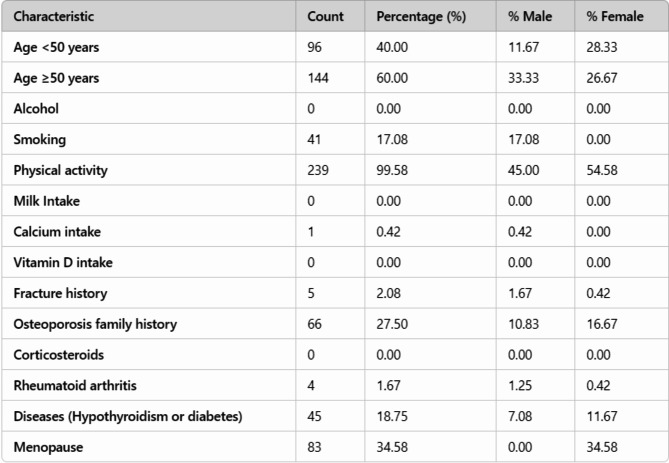
Demographic analysis of the dataset

The demographic characteristics of the 240 patients were analysed to assess the prevalence of the various factors that can potentially affect bone health (Fig. 4). Among the participants, 40.0% were under the age of 50, while 60.0% were aged 50 and above. Notably, there were no reported cases of alcohol consumption or milk intake among the subjects, and only 0.42% reported calcium intake. Smoking was prevalent in 17.08% of the patients, while a significant majority (99.58%) engaged in some form of physical activity. A fracture history was reported in 2.08% of participants, and 27.5% had a family history of osteoporosis.

Gender-specific analysis revealed that males accounted for 11.67% of participants under 50 years of age and 33.33% of those aged 50 years and older. Among smokers, 17.08% were male, whereas physical activity was more balanced with 45.0% male participation compared to 54.58% female. Furthermore, 34.58% of the female participants reported menopause. Lastly, 18.75% of patients had comorbid conditions such as hypothyroidism or diabetes, with 7.08% being male and 11.67% female. Overall, these demographics highlight the need for tailored interventions for different age and gender groups concerning bone health.

### network and architecture

**Fig. 5:**
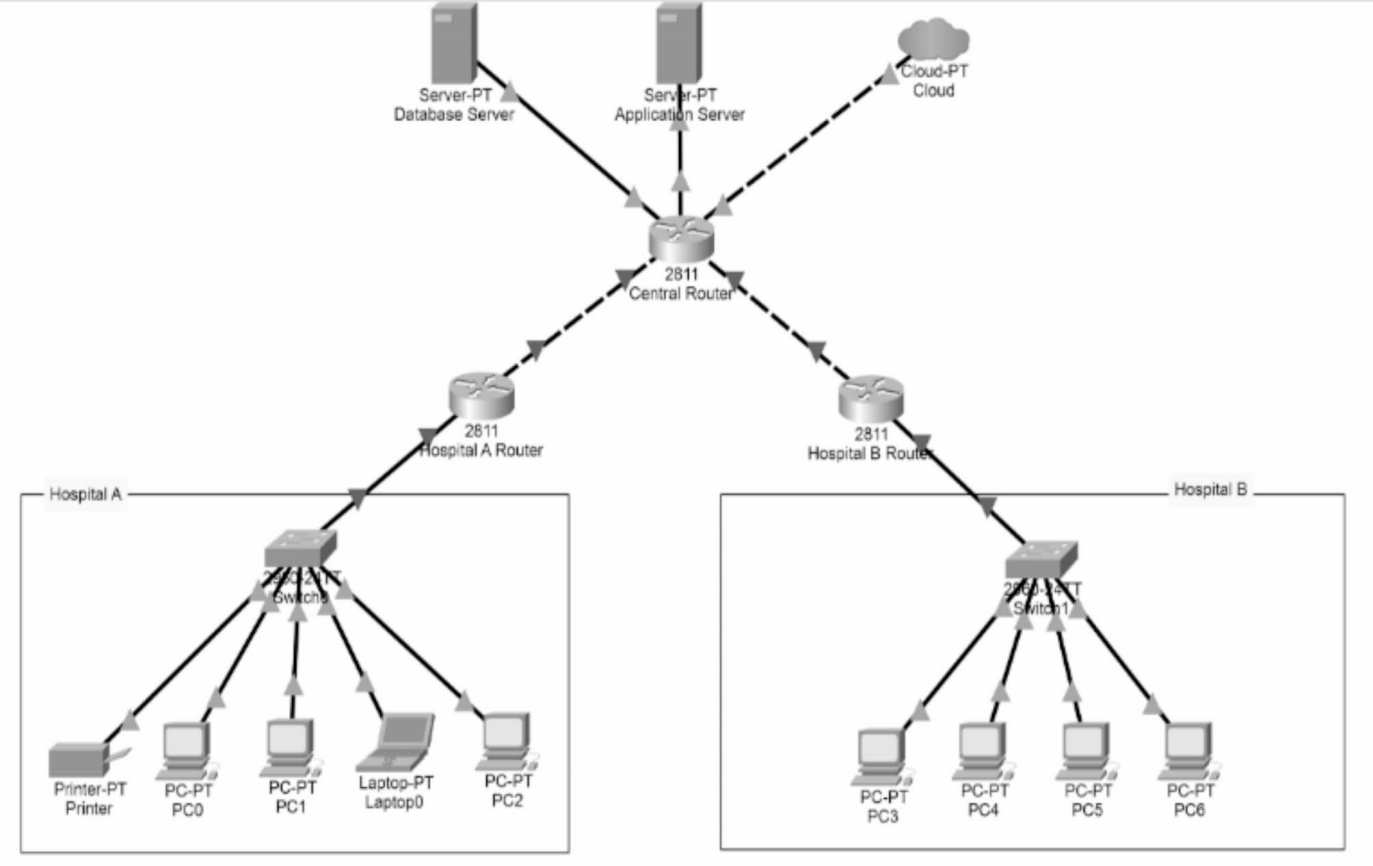
Recommended architecture

The architecture outlined in Fig. 5 demonstrates the scalability and reliability of the proposed system for real-time osteoporosis detection across multiple healthcare facilities. This architecture reflects a robust infrastructure capable of supporting high-traffic environments and secure data processing across a distributed network.

Each healthcare facility will be equipped with client devices like computers or tablets that gather patient data. These devices connect to a local area network (LAN) via a switch, allowing internal communication. When transmitting patient data, the data is routed through the facility’s router, which connects to the internet. To ensure secure transmission, the data must be encrypted through a Virtual Private Network (VPN) that safeguards it as it travels over the internet to the centralized servers.

The central servers, hosted either on-premises or in the cloud, may then process the incoming data by running the osteoporosis detection model. Once the model generates predictions, these results are sent back to the healthcare providers in a timely manner, ensuring they have access to accurate information for informed decision-making.

In a production environment, multiple servers will host the application, with an Application Load Balancer (ALB) integrated to manage traffic. The ALB dynamically distributes incoming requests to the available servers, ensuring that the system can handle high traffic from several healthcare facilities without overloading any single server. This is crucial in environments where multiple healthcare professionals may simultaneously need to submit data for analysis, much like the need for timely and accurate diagnoses in clinical settings.

Furthermore, robust network security measures, such as firewalls and intrusion detection systems (IDS), should be implemented to protect the application servers and database servers from unauthorized access.

## Limitations

A. The study was conducted on a relatively small sample size, which may limit the generalizability of the findings.
B. All data was collected from a single clinical center, which might introduce bias related to regional or institutional practices.
C. Although the model showed high accuracy, further validation on larger, multi-center datasets is necessary to confirm its robustness and applicability.
D. The study focused solely on calcaneus QUS measurements and did not include other anatomical sites that may also provide critical insights into bone health.

## Conclusion

The predictive model developed in this study serves as a valuable **preliminary screening tool** for identifying individuals at high risk of osteoporosis, reducing the need for costly and limited-access DEXA scans. By using widely available patient health data, the model effectively narrows the individuals truly requiring further diagnostic confirmation. This model has several applications in the **medical field**, particularly in resource-limited settings, where early detection is crucial to prevent and manage osteoporosis proactively.

While the model can assist in improving osteoporosis detection, it is essential to highlight its role as a **supplement to doctors** rather than a replacement. It provides support in identifying risk factors, but the final diagnosis should always involve clinical evaluation by certified healthcare professionals. The model’s **feature importance analysis** has revealed that variables such as T-score, age, BMI, and menopause status are crucial for prediction, thus guiding practitioners on the **key features to monitor** during patient screening.

This model could be enhanced through continuous refinement, incorporating new clinical features, and expanding its application to diverse populations. With proper validation, it holds the potential to transform osteoporosis screening, making it more **accessible and cost-effective** for healthcare settings globally.

## Data Availability

The dataset supporting the findings of this study, “Knee X-ray Osteoporosis Database,” is available on Mendeley Data under the DOI: 10.17632/fxjm8fb6mw.2. The dataset includes demographic details, clinical history, and Quantitative Ultrasound (QUS)-derived T-scores for osteoporosis diagnosis.

## Abbreviations

QUS: Quantitative Ultrasound
DXA: Dual–energy X–ray Absorptiometry
BMD: Bone Mineral Density
WHO: World Health Organization
ML: Machine Learning
BMI: Body Mass Index
LAN: Local Area Network
VPN: Virtual Private Network
ALB: Application Load Balancer
IDS: Intrusion Detection Systems
